# The Prevalence of Orthorexia Nervosa in Polish and Lebanese Adults and Its Relationship with Sociodemographic Variables and BMI Ranges: A Cross-Cultural Perspective

**DOI:** 10.3390/nu12123865

**Published:** 2020-12-17

**Authors:** Anna Brytek-Matera, Hala Sacre, Anna Staniszewska, Souheil Hallit

**Affiliations:** 1 Institute of Psychology, University of Wroclaw, Dawida 1, 50-527 Wroclaw, Poland; 2 Faculty of Medicine and Medical Sciences, Holy Spirit University of Kaslik (USEK), Jounieh, Lebanon; hala.sacre@opl.org.lb (H.S.); souheilhallit@hotmail.com (S.H.); 3 Department of Experimental and Clinical Pharmacology, Medical University of Warsaw, 02-091 Warsaw, Poland; astaniszewska@wum.edu.pl; 4 INSPECT-LB: Institut National de Santé Publique, Épidémiologie Clinique et Toxicologie-Liban, Beirut, Lebanon

**Keywords:** orthorexia nervosa, eating behavior, body mass index, sex, cross-cultural study

## Abstract

The prevalence of orthorexia nervosa (ON) appears to be increasing, and more research into its cross-cultural aspects is required to provide culturally appropriate psychological treatment. Until now, there has been relatively little research published about ON across cultures. Therefore, the objectives of the present study were to determine: (1) the prevalence of ON in Polish and Lebanese adults and (2) the association between ON and sociodemographic variables and Body Mass Index (BMI) in two culturally different samples. One thousand two hundred and sixty-two adults participated in the present study (*N*_Poland_ = 743 adults; *N*_Lebanon_ = 519 adults). The Düsseldorf Orthorexia Scale and the Eating Habits Questionnaire were used in the present study. Information about age, sex, anthropometry, and marital status was obtained from all participants as well. The Polish sample had an ON prevalence rate of 2.6%, while the Lebanese sample had an ON prevalence rate of 8.4%. No significant correlation was found between ON and age in both samples. A statistically significant difference was found between marital status and country on ON, with the highest mean score seen among Lebanese singles. In Lebanon, having a low of BMI ≤ 25 kg/m^2^ compared to a high BMI was significantly associated with lower ON tendencies, while this association was not significant among Polish participants. This study was the second to focus on the prevalence of ON in Western and non-Western countries and its association with sociodemographic characteristics and BMI ranges. Knowledge about ON and its correlates in diverse populations may inform the design of culturally tailored behavior change interventions and the development of culturally appropriate tools in various groups to improve their dietary patterns.

## 1. Introduction

Over recent decades, healthy eating has become progressively idealized in our society [1]. Recent years have witnessed increasing popularity in a new trendy dietary strategy (“clean eating”) widely propagated through social media [2]. “Clean eating”, which emphasizes the consumption of healthy, “pure” foods, may reflect susceptibility to a pathological fixation with healthy eating [2,3]. It typically includes elements, such as eating local, “real” (non-processed), organic, plant-based, home-cooked foods, as well as extreme strategies, such as eliminating gluten, grains, or dairy [2]. “Clean eating” is linked to health-related attitudes and behaviors and could have negative health consequences (e.g., amenorrhea, osteoporosis, bone fractures, irregular heartbeats, difficulties concentrating) [2]. A clinically meaningful, pathological obsession (or excessive preoccupation) with eating only healthy, “clean” and “pure” foods, as well as disturbing thoughts and excessive worrying regarding healthy dietary intake [4,5], has been called orthorexia nervosa (ON). This fixation on the purity of food (quality) is the main feature of ON. ON is characterized by obsessive behaviors and preoccupation with healthy nutrition that includes rigidly following a restrictive “healthy” diet (that the individual believes to be healthy and pure) with strict avoidance of food considered unhealthy. The intrusive, food-related thoughts generate emotional consequences (severe distress, feelings of guilt, shame) and self-punishment of non-adherence to self-imposed dietary rules as well as psychosocial and physiological (e.g., malnutrition and weight loss) impairments [6,7]. As in the case of extreme “clean eating” [2], ON could have negative health consequences that resemble those of an eating disorder (e.g., difficulties concentrating, amenorrhea, depression) [8,9,10]. Additionally, the need to omit certain food groups without justification may contribute to disordered eating attitudes and behaviors [11], and/or mask already existing anorexia nervosa (AN) or be a prelude to AN [12].

Numerous researchers have reported the prevalence of ON, with values varying considerably according to the measure used to evaluate the same construct (e.g., ORTO-15 test, the Düsseldorf Orthorexia Scale), the selected population (e.g., clinical versus non-clinical samples), and the nationality [13]. The prevalence of ON ranges from 1% in the general U.S. population [14] to much higher percentages, particularly among university students worldwide, e.g., 6.6% in a sample of Polish students [15], 8% in a sample of U.S. students [16], and 10.5% in a sample of Spanish students [13]. It has been suggested that the vast inconsistency was mainly due to the difference of instruments used, but not due to cultural differences [14].

ON is conceptualized as being linked to cultural concepts of health pervasive in contemporary Western societies [17]. To date, the majority of empirical research evaluating the prevalence of ON has been conducted in European countries, such as Italy, Hungary, Poland, Turkey, Germany, and Spain [12,18,19,20,21]. However, little is known about the prevalence and clinical correlates of ON in non-Western cultures [17]. ON has been poorly studied in Western and East Asia; to the best of our knowledge, one study has been conducted in Korea [22], two studies in China [17,23], and three in Lebanon [24,25,26].

Regarding ON and sociodemographic variables, sex has been generally found to be unrelated to ON [27,28,29], whereas findings between ON and age were controversial. Some studies found no relationship [28,30], while others found a weak positive relationship [31] or weak negative relationship [27]. According to Strahler et al. [10], the impact of age on ON seems negligible.

Results from recent studies have yielded inconsistent evidence on the relationship between ON and Body Mass Index (BMI). A large number of studies found no link between ON and BMI [16,23,25,30,32,33]. Others suggested positive [19,21,34,35] or negative [31,36] relationship between ON and BMI. Godefroy, Trinchera, and Dorard [37] concluded that the validated structural model globally suggests that ON is not typically related to high or low BMI. Their findings [37] have demonstrated that ON dimensions are only marginally related to BMI.

The prevalence of ON appears to be increasing, and more research into its cross-cultural aspects is required to provide culturally appropriate psychological treatment. Until now, there has been relatively little research published about ON across cultures [21,24,38]. Therefore, the objectives of the present study were to determine: (1) the prevalence of ON in Polish and Lebanese adults and (2) the association between ON and sociodemographic variables and BMI in two culturally different samples. Based on a recent cross-cultural study [24], we hypothesize that: (H1) the prevalence of ON will be higher in Lebanese sample compared with the Polish sample, and (H2) ON will be related with BMI categories in both Polish and Lebanese populations.

## 2. Materials and Methods

### 2.1. Participants and Study Design

The Polish sample consisted of 743 adults (571 women and 172 men) with a mean age of 24.80 years old (SD = 6.76). The Lebanese sample included 519 adults (283 women and 236 men) with a mean age of 35.83 years old (SD = 14.48). In total, 1262 adults participated in the present study.

In Poland, convenience sampling recruited adults to participate in an online survey. Participants received notice about the research at various institutions (e.g., universities, companies) with the announcement including the online link to the study. Interested individuals were invited to visit a website that directed them to the consent form, information form (objective of the study, anonymity, voluntariness of consent to research), and questionnaires. All participants offered their informed consent before starting the survey (by ticking a respective box at the first page of the online survey) and responded voluntarily to the survey. Of the 856 individuals who started the questionnaires, 743 (86.79%) completed the survey. In Lebanon, data were collected using paper questionnaires or face-to-face interviews. The Lebanese sample was recruited from seven community pharmacies chosen randomly from a list provided by the Lebanese Order of Pharmacists, the official pharmacists’ association in Lebanon. Of the 700 questionnaires distributed, 519 (74.14%) were completed and collected back. In total, 81.10% participants who were eligible completed the study. Participants received no financial compensation for enrolling in the study.

The present study has been approved by the local research ethics committees in the two countries (SWPS University of Social Sciences and Humanities Human Research Ethics Committee, No. WKEB59/05/2019; Psychiatric Hospital of the Cross, No. HPC-13-2019). All procedures performed in our study were in accordance with the 1964 Helsinki declaration (adopted by the 18th World Medical Association General Assembly, Helsinki, Finland) and its later amendments or comparable ethical standards.

### 2.2. Measures

#### 2.2.1. Translation Procedure of the Questionnaire Used in a Lebanese Sample

A forward and backward translation was performed for all the scales by two translators, one for the translation from English into Arabic, and the other for the back-translation. During the forward translation phase, the principal emphasis was to reach equality between the English and Arabic versions while using a comprehensible vocabulary. The Arabic form was revised by an expert committee composed of the original translator, one psychiatrist, and two psychologists. The same process was used in the back-translation from Arabic into English. Discrepancies between the original and translated English versions were resolved by consensus. A pilot study was conducted on 20 persons to ensure that the questions are well understood; no significant changes were made to the Arabic version subsequently, thus, the results were included in the final dataset.

#### 2.2.2. ON

Two measures were used to assess ON: the Düsseldorf Orthorexia Scale (DOS) [39] and the Eating Habits Questionnaire (EHQ) [40]. The DOS measures the prevalence of ON. It consists of 10 items scored on a four-point Likert scale (1 = never, 2 = rarely, 3 = often, and 4 = always). Higher points indicate more pronounced orthorexic behavior. The maximum score is 40. A score of 30 or higher indicates the presence of ON. A score between 25 and 29 identifies a risk of developing ON, while a total score of less than 25 demonstrates the absence of ON [39]. The DOS shows good internal consistency (Cronbach’s alpha = 0.84) and good retest reliability (*r* = 0.67 to 0.79, *p* = 0.001 between three reference dates) [39]. In the present study, the Polish version (PL-DOS) [15] and the Lebanese version of the DOS [41] were used (Cronbach’s α_Poland_ = 0.834 and Cronbach’s α_Lebanon_ = 0.896).

The EHQ is a 21-item tool that assesses the cognitions, behaviors, and feelings related to an extreme focus on healthy eating, called ON. The EHQ is rated on a four-point Likert scale (1 = false, not at all true, 2 = slightly true, 3 = mainly true, 4 = very true). The subscales of the EHQ capture: (1) knowledge of healthy eating (e.g., “I know more about healthy eating than do other people”), problems associated with healthy eating (e.g., “I spend more than three hours a day thinking about healthy food”), and (3) feeling positively about healthy eating (e.g., “I feel in control when I eat healthily”). Preliminary evidence suggests that the EHQ is reliable, with the three subscales showing strong internal consistency (Cronbach’s α = 0.82, 0.90, and 0.86, respectively) [40]. In the present study, the Polish version [41] and the Arabic version of the EHQ were used (the Cronbach’s α values of the three subscales were: knowledge of healthy eating: Cronbach’s α_Poland_ = 0.868 and Cronbach’s α_Lebanon_ = 0.904, problems associated with healthy eating: Cronbach’s α_Poland_ = 0.834 and Cronbach’s α_Lebanon_ = 0.809 and feeling positively about healthy eating Cronbach’s α_Poland_ = 0.774 and Cronbach’s α_Lebanon_ = 0.796).

#### 2.2.3. Sociodemographic Variables

The questionnaire used gathered information about age, sex, anthropometry (weight and height), and marital status. In both countries, height and weight were self-reported and used to calculate BMI. BMI was categorized according to the World Health Organization (WHO) recommendations [42]: underweight <18.5 kg/m^2^, normal weight between 18.5 and 24.9 kg/m^2^, overweight between 25 and 29.9 kg/m^2^, and obesity >30 kg/m^2^.

### 2.3. Statistical Analysis

SPSS version 25 (IBM, Armonk, NY, USA) was used to perform data analysis. Data were screened for missing values and missing data deleted from the analyses (participants who did not have complete data were omitted). The missing values were not replaced since they did not account for more than 5% of the entire database. For the reduction of data, an exploratory factor analysis (EFA) was first executed on sample 1. The Kaiser–Meyer–Olkin (KMO) index and Bartlett’s Chi-square test of sphericity confirmed the sample’s adequacy. Factors retained corresponded to those with an Eigenvalue >1. A two-way analysis of variance (ANOVA) was used to compare the mean differences between two independent variables (country and sex) by considering the partial eta squared as an effect size. Partial correlation, including sex as a covariate, was used for linear correlation between continuous variables (in one country). Fisher’s Z was used to compare correlation coefficients, and X2 and the Fisher exact tests were used to compare categorical variables. A partial eta squared of │0.01–0.05│indicated a small effect, while values of│0.06–0.13│and >│0.14│ indicated moderate and large effects, respectively [43]. A value of *p* < 0.05 was considered significant.

The G*Power 3.1.9.2 software [44] calculated a minimal sample of 210 participants to detect sex-specific differences between countries, with a 95% power, a 5% error, and a 0.25 effect size (set arbitrarily in the absence of studies comparing orthorexic tendencies and behaviors between Poland and Lebanon).

## 3. Results

### 3.1. Sociodemographic Characteristics of the Sample Population

Table 1 shows the sociodemographic details of the Polish and the Lebanese samples. Higher mean age and BMI were significantly found in the Lebanese sample as compared with the Polish one. A higher proportion of females and being single were significantly found in the Polish versus the Lebanese sample.

### 3.2. Factor Analysis of the DOS and EHQ Scales in a Lebanese Sample

The total sample (*n* = 519) was used for the factor analysis of the DOS and EHQ scales; all items of both scales were extracted and yielded a two-factor solution for the DOS scale (Factor 1: adherence to strict nutrition rules and emotional symptoms; Factor 2: Obsession in healthy food) and a three-factor solution for the EHQ scale (Factor 1: problems; Factor 2: knowledge; Factor 3: feelings) with Eigenvalues >1. The KMO and the *p*-value of the Bartlett’s sphericity test ensured adequacy of both scales. Moreover, the αCronbach of both scales were excellent (Table 2 and Table 3).

### 3.3. Prevalence of ON: A Cross-Cultural Comparison

Table 4 provides an overview of the total sample and sex-specific means and standard deviations of the DOS sum score. The mean DOS score in the Polish sample was 17.7 ± 5.1; 90.4% were categorized as having no risk of ON, 7.0% at risk of developing ON, and 2.6% having ON. In the Lebanese sample, the mean DOS score was 20.0 ± 7.0, with 74.1% subjects with no risk of ON, 17.5% at risk of developing ON, and 8.4% having ON.

A two-factor ANOVA was conducted to examine the effect of country and sex on DOS scores after adjusting for covariates (age, BMI, and marital status). Analyses of the DOS sum score revealed a country effect, F(1,1195) = 41.63, *p* < 0.001, η^2^ = 0.034, with higher levels in Lebanon sample than the Polish one (20.22 vs. 17.45). The sex and the interaction effects were not statistically significant, with F(1,1195) = 3.50, *p* = 0.061, η^2^ = 0.003 and F(1,1195) = 0.065, *p* = 0.799, η^2^ = 0.00001, respectively.

### 3.4. Correlates of ON: Cognitions, Behaviors, and Feelings Related to an Extreme Focus on Healthy Eating

Table 5 shows the relationship between ON and knowledge of healthy eating, problems associated with healthy eating, and feeling positively about healthy eating in both samples. The three EHQ subscales were positively correlated with DOS in the Polish adults and negatively correlated with DOS in the Lebanese adults.

A two-factor ANOVA was also conducted to examine the effect of country and sex on the EHQ subscales after adjusting for covariates (age, BMI, and marital status). Analyses of the EHQ problems score revealed a country effect, with higher levels in the Lebanese sample than the Polish one (36.66 vs. 16.28). The sex and the interaction effects were not statistically significant. Analyses of the EHQ knowledge score revealed a country effect, with higher levels in the Lebanese sample than the Polish one (14.82 vs. 8.92). The sex effect was not statistically significant; finally, the interaction sex by country effect showed statistical significance. Analyses of the EHQ feelings score revealed a country effect, with higher levels in the Lebanese sample than the Polish one (11.27 vs. 8.58). The sex effect was not statistically significant; finally, the interaction sex by country effect showed statistical significance (Table 6).

### 3.5. Correlates of ON: Sociodemographic Variables and Body Mass Index

No significant correlation was found between age and DOS scores in both samples. A statistically significant difference was found between marital status and country on the DOS, with the highest mean score seen among Lebanese singles, with no significance found in terms of the interaction between marital status by country (all *p* = 0.991, Table 7).

Finally, a two-way ANOVA examined the effect of BMI categories on the DOS scores and showed a statistically significant main effect of the country on the DOS sum score (η^2^ = 0.004), with the highest levels observed in Lebanese participants compared to Polish ones (19.19 vs. 17.52). The interactions between the effect of BMI categories and country on DOS scores were significant (all *p* = 0.008, Table 7).

**Figure 1:**
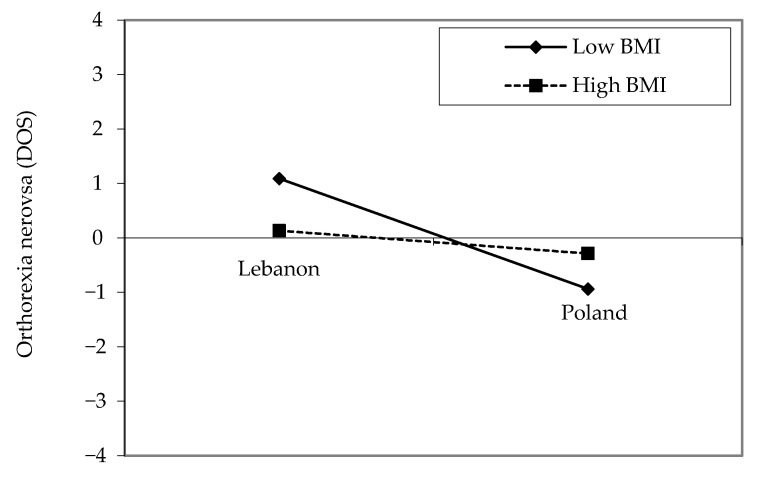
Plots of the two-way interaction effect between country and Body Mass Index. Note: In this figure, standardized values were used.

Because the interactions between the effect of dichotomous BMI variable and country on DOS scores was significant, we assessed whether the association between BMI (high versus low) and DOS was moderated by the country. Analyses of the DOS score revealed a country effect, F(1,1195) = 229.65, *p* < 0.001, η^2^ = 0.024, with higher levels in Lebanon sample than the Polish one (20.15 vs. 17.77). The effect of the dichotomous BMI variable was not statistically significant, with F(1,1195) = 1.11, *p* = 0.292, η^2^ = 0.001; finally, the interaction sex by dichotomous BMI effect showed statistical significance F(1,1195) = 6.88, *p* = 0.009, η^2^ = 0.006. In Lebanon, having a low BMI ≤ 25 kg/m^2^ compared to high BMI was significantly associated with lower DOS scores (B = −1.632; *p* = 0.025; 95% CI −3.061–−0.203; η^2^ = 0.011), whereas this association was not significant among Polish participants (Figure 1).

## 4. Discussion

Our study included two ON-associated objectives. The first was to assess the country-specific prevalence of ON. The prevalence of ON was higher in the Lebanese sample in comparison to the Polish one (8.4% versus 2.6%) (H1 was confirmed). Our findings are consistent with a recent study [24] presenting higher levels of ON among Lebanese adults comparing with the German adults (8.4% versus 4.9%). Additionally, in the present study, more Lebanese adults than Polish adults were at risk of developing ON and had ON. This difference was driven by women. A recent Lebanese study [26] has shown that the female sex was associated with higher ON tendencies and behaviors.

According to the WHO (2010), the Middle East region is classified as a region in a nutritional transition stage. Ten years ago [45], Lebanon’s nutrition profile was proposed as being in early nutrition transition. Countries included in this category (e.g., Egypt, Jordan, Syrian Arab Republic) were characterized by moderate levels of overweight and obesity, moderate levels of undernutrition in specific population and age groups, and widespread micronutrient deficiencies. Nowadays, the nutrition transition is characterized by a rapid change of dietary intake from traditional, diverse, and balanced diets to more “westernized” diets (specifically a high consumption of “harmful” foods and low consumption of “protective” foods) [46]. The previous research has demonstrated that nutritionally related health patterns and Western lifestyle trends are being adopted in the Middle East [47]. Haddad et al. [26] stated that the food- and nutrition-related behaviors leading to unhealthy eating behaviors among the Lebanese population might result from the media effect concerning healthy eating (false or misleading messages or advertisements may be misinterpreted and adversely affect eating behaviors as well). Moreover, the awareness-raising about healthy nutrition and health-related behaviors are observed among the Lebanese population and can contribute to the growing concerns about dietary patterns [26] as well as influence preferences toward too healthy foods. Research has shown that nutritional awareness has a direct effect on diet quality [48]. In individuals with ON, it could influence the amount of “healthy” food consumption with strict avoidance of food considered unhealthy. Indeed, following the positive influence the nutrition education program had on some Lebanese schools, the Ministry of Education and Higher Education released a decree that reinforced the sale of healthy food items in schools while forbidding the sale of energy-dense supplements/foods (sweetened drinks containing <30% fruit juice, soft and energy drinks, all meat products, fried foods, sugar-coated fruits, candies, chewing gum, and sweets containing cream and or syrup) [49]. The Ministry of Public Health also implemented the “Food-Based Dietary Guideline Manual” for healthy eating promotion in Lebanese adults [49]. These steps might have contributed in one way or another to the development of ON tendencies and behaviors in Lebanon; however, this remains to be confirmed by future studies in the country.

Since an Arab dietary pattern as such is yet to be ascertained, as typical food selection (consumption practices varying widely from country to country) [47], it is difficult to compare the Arab dietary pattern with the Polish one. A recent study [50] about the westernization of dietary patterns among young Polish females have found three dietary patterns: “prudent,” “Western,” and “sweets and alcoholic beverages.” Among the Polish sample, the extent of diet westernization was observed [50]. Moreover, the link between dietary patterns and ON exists [51]. Polish students who consumed high-sugar products and snacks and presented fatty products and dressings patterns most often were less likely to display ON tendency [51], in contrast with students who consumed fresh products and nuts and were more likely to develop ON tendency. We can assume that, nowadays, the influence of westernization on dietary patterns in the Lebanese population could be more important than it is in the Polish one. However, it requires empirical verification. Two studies conducted among Lebanese [52,53] have shown four and three dietary patterns, respectively. The four dietary patterns included western (fast food, including pies and pizza, fast-food sandwiches, fried potatoes, regular soda, bottled juices, meat and poultry, cured meats, nuts and seeds, refined grains, mayonnaise, ice-cream, and sweets), traditional Lebanese (fruit, vegetables, burghul, legumes, olives, whole-fat dairy, starchy vegetables, fats and oils, eggs), prudent (primarily whole bread, low-fat dairy, light soda), and fish and alcohol [52,53]. The three dietary patterns consisted of fast food/dessert (high intake of fast food sandwiches including hamburgers, shawarma, falafel, pizzas, pies, desserts, carbonated beverages and juices, and mayonnaise), traditional Lebanese dietary pattern (dairy products, olives, fruits, legumes, grains, eggs, vegetable oil, dried fruits, and traditional sweets), and high protein (high intake of fish, chicken, meat, and low-fat dairy products). It could be hypothesized that in cultures that have always highly valued healthy eating as being the basis of their way of living [13], the process of westernization of dietary patterns would be different from that of cultures, which have distinct ideas regarding the maintenance of healthy eating pattern.

Our second purpose was to evaluate the association between ON and sociodemographic variables and BMI ranges in two culturally different samples. Our findings are consistent with previous studies showing no relationship between ON and age in both samples [28,30]. Strahler et al. [24] achieved the opposite results: in German adults, ON was negatively related to age, while this association was positive in Lebanese adults. That indicates the inconsistent evidence on this abovementioned relationship [24].

In our study, a significant difference was observed between marital status and country on the DOS, with the highest mean score seen among Lebanese singles, in contrast with a previous study conducted between Germany and Lebanon that showed no association between marital status and ON [24]. This difference might be due to other factors not taken into consideration in this study, such as body dissatisfaction. We also hypothesize that single people have more time to focus on healthy eating compared to married people who focus on other tasks (raising kids, house chores, etc.). The association between marital status and ON remains blurry, with more studies needed to resolve this controversy.

A positive relationship between ON and cognitions, behaviors, and feelings related to an extreme focus on healthy eating was found among Polish adults and is consistent with the previous results [16,24]. This finding could indicate that in the Polish sample the DOS and the EHQ measure the same construct. Opposite results were observed in Lebanese individuals, which indicates the need for more research about the ON measure in Lebanon.

Despite its strengths (i.e., sample size), this study has some limitations. First, the results of the convenience sampling cannot be extrapolated to the general population. We performed a cross-cultural study among university students in Poland mostly and among persons coming to pharmacies in Lebanon (the two groups were composed differently; the mean age of the participants differed across countries). Second, it is noteworthy that the Polish adults participated online, whereas the Lebanese adults had an in-person interview (data collection methodology was different between the two countries). Third, sample sizes were unequal across sex (lower numbers of men in both groups). Fourth, subjectively measured body mass index (possibility of inaccurate estimation of body weight and height). Fifth, although the EHQ measures the cognitions, behaviors, and feelings related to ON, the use of the EHQ cannot discriminate ON from non-ON. Sixth, religion was not assessed in the Lebanese sample, where up to two-thirds of individuals are Muslim. Although the data were not collected during a fasting period (Ramadan or Easter), fasting as part of a devout religious practice may have affected the EHQ and DOS scores. The latter idea is thought not to affect the results since all food products available on the Lebanese market are labeled “Halal” and both Christians and Muslims eat the “Halal” products (except for pork and alcohol products). Seventh, although the item formats, rating scales, of the Lebanese and Polish versions of the DOS and EHQ scales were suitable, our results should be interpreted with caution regarding the scores categories. Eighth, detailed information on dietary patterns in Polish and Lebanese individuals should be available for providing better knowledge of the nutrition sample profiles. Finally, a residual confounding bias might be present since not all factors associated with ON were taken into consideration during this study.

## 5. Conclusions

This study was the second to focus on the prevalence of orthorexia nervosa in Western and non-Western countries, and its association with sociodemographic characteristics and BMI ranges. Knowledge about ON and its correlates in diverse populations may inform the design of culturally tailored behavior change interventions [54] and the development of culturally appropriate tools in various groups to improve their dietary patterns.

Further longitudinal research on ON is needed to examine the causal relationship between ON and health-related and eating-related behaviors in Polish and Lebanese adults. Moreover, characterizing nutrition profiles of both Polish and Lebanese samples may help in developing psychoeducational programs and nutritional interventions among individuals with different dietary patterns and having ON or being at risk or not of developing ON. The socioeconomic status and education level could be included in sociodemographic variables in future studies to assess whether higher socioeconomic status and higher education level are associated with ON.

## Figures and Tables

**Table 1:** Comparison of sociodemographic characteristics in both samples.

	Polish Adults (*n* = 743)	Lebanese Adults (*n* = 519)	Degree of Freedom	Pearson Chi-Square, *p*-Value	Effect Size
	Frequency (%)	Frequency (%)			
Sex					
Male	172 (23.1%)	222 (44.0%)		60.27, *p* < 0.001	V_Cramer_ 0.220
Female	571 (76.9%)	283 (56.0%)			
Marital status					
Single	653 (87.9%)	264 (51.7%)		202.16, *p* < 0.001	V_Cramer_ 0.402
Married	90 (12.1%)	247 (48.3%)			
	Mean ± SD	Mean ± SD		Student’s t, *p*-value	
Age (years)	24.80 ± 6.76	35.83 ± 14.48	1255	18.10, *p* < 0.001	Cohen’s d 0.976
Body Mass Index (kg/m^2^)	22.96 ± 3.66	24.45 ± 4.34	1214	6.42, *p* < 0.001	Cohen’s d 0.371

Some variables’ values might not add up to the total sample size because of missing values.

**Table 2:** Factor analysis of the Düsseldorf Orthorexia Scale (DOS) items in a Lebanese Sample.

Factor Analysis of the DOS Items According to the Promax Rotation in Lebanon.					
Question	Item Number	Factor 1	Factor 2	h2 Communalities	Item-Factor Correlation *
I have the feeling of being excluded by my friends and colleagues due to my strict nutrition rules	7	0.982		0.720	0.612
I try to avoid getting invited over to friends for dinner if I know that they do not pay attention to healthy nutrition	4	0.825		0.586	0.646
My thoughts constantly revolve around healthy nutrition and I organize my day around it	8	0.718		0.670	0.769
I find it difficult to go against my personal dietary rules	9	0.686		0.634	0.734
If I eat something I consider unhealthy, I feel really bad	6	0.613		0.633	0.768
I feel upset after eating unhealthy foods	10	0.575		0.533	0.684
Eating healthy food is more important to me than indulgence/enjoying the food	1		0.917	0.724	0.663
I have certain nutrition rules that I adhere to	2		0.906	0.713	0.670
I can only enjoy eating foods considered healthy	3		0.749	0.611	0.692
I like that I pay more attention to healthy nutrition than other people	5		0.632	0.650	0.750
Percentage of variance explained	64.74	51.91	12.83		
Cronbach’s alpha	0.896	0.870	0.836		

* *p* < 0.001 for all correlations; KMO = 0.898; Bartlett’s test of sphericity *p* < 0.001; h2 communalities indicate the total amount of variance a variable shares with all other variables in the factor analysis.

**Table 3:** Factor analysis of the Eating Habits Questionnaire (EHQ) items in a Lebanese Sample.

Factor Analysis of the EHQ Items According to the Promax Rotation in Lebanon.						
Question	Item Number	Factor 1	Factor 2	Factor 3	h2 Communalities	Item-Factor Correlation *
I turn down social offers that involve eating unhealthy food.	2	0.452			0.531	0.812
I follow a diet with many rules.	4	0.602			0.567	0.795
I am distracted by thoughts of eating healthily.	6	0.738			0.552	0.8001
I only eat what my diet allows.	7	0.722			0.571	0.838
My healthy eating is a significant source of stress in my relationships.	8	0.828			0.696	0.834
My diet affects the type of employment I would take.	10	0.782			0.642	0.813
In the past year, friends or family members have told me that I’m overly concerned with eating healthily.	13	0.705			0.557	0.866
I have difficulty finding restaurants that serve the foods I eat.	14	0.552			0.536	0.832
Few foods are healthy for me to eat.	16	0.466			0.720	0.773
I go out less since I began eating healthily.	17	0.677			0.545	0.846
I spend more than three hours a day thinking about healthy food.	18	0.737			0.585	0.844
I follow a health-food diet rigidly.	20	0.738			0.565	0.856
I am more informed than others about healthy eating.	1		0.775		0.603	0.698
The way my food is prepared is important in my diet.	3		0.799		0.656	0.702
My eating habits are superior to others.	5		0.658		0.599	0.787
My diet is better than other people’s diets.	11		0.751		0.595	0.832
I prepare food in the most healthful way.	21		0.668		0.516	0.764
I have made efforts to eat more healthily over time.	9			0.566	0.535	0.595
I feel in control when I eat healthily.	12			0.677	0.642	0.749
Eating the way I do gives me a sense of satisfaction.	15			0.707	0.617	0.629
I feel great when I eat healthily.	19			0.635	0.509	0.493
Percentage of variance explained	58.76	45.77	8.22	7.77		
Cronbach’s alpha	0.969	0.969	0.893	0.807		

* *p* < 0.001 for all correlations; KMO = 0.944; Bartlett’s test of sphericity *p* < 0.001. h2 communalities indicate the total amount of variance a variable shares with all other variables in the factor analysis.

**Table 4:** Comparison of orthorexia nervosa (ON) prevalence among Polish and Lebanese samples.

Variable	Polish Adults (*n* = 743,571 Women)	Lebanese Adults (*n* = 519,283 Women)
Düsseldorf Orthorexia Scale (DOS) M ± SD	17.7 ± 5.1 f: 17.8 ± 4.8 m: 17.2 ± 6.0	20.0 ± 7.0 f: 20.4 ± 7.1 m: 19.6 ± 6.8
DOS risk categories		
Having ON (DOS ≥ 30) *n*, % within sex	f: 12 (2.1%) m: 7 (4.1%)	f: 26 (9.3%) m: 17 (7.7%)
At risk of developing ON (DOS 25–29) *n*, % within sex	f: 42 (7.4%) m: 10 (5.8%)	f: 55 (19.6%) m: 33 (15.0%)
Having no risk of ON (DOS ≤ 24) *n*, % within sex	f: 517 (90.5%) m: 155 (90.1%)	f: 200 (71.2%) m: 170 (77.3%)

Note: M: mean, SD: standard deviation; f: female; m: male.

**Table 5:** Correlation between ON and cognitions, behaviors, and feelings related to an extreme focus on healthy eating in Polish and Lebanese samples.

	Düsseldorf Orthorexia Scale (DOS) *r*, *p*	
	Polish Adults	Lebanese Adults
Knowledge of healthy eating	0.396, *p* < 0.001	−0.635, *p* < 0.001
*Z*, *p*	−20.38; *p* < 0.001	
Problems associated with healthy eating	0.493, *p* < 0.001	−0.633, *p* < 0.001
*Z*, *p*	−22.43; *p* < 0.001	
Feeling positively about healthy eating	0.325, *p* < 0.001	−0.628, *p* < 0.001
*Z*, *p*	−18.75; *p* < 0.001	

**Table 6:** Two-factor ANOVA results of the country and sex effect on the EHQ subscales scores.

	F Value	*p*	Partial Eta Squared η^2^
**Dependent variable: EHQ problems score**			
Country (Poland vs. Lebanon *)	1766.35	<0.001	0.598
Sex (females vs. males *)	0.334	0.563	0.00001
Interaction sex-country	2.501	0.114	0.002
**Dependent variable: EHQ knowledge score**			
Country (Poland vs. Lebanon *)	543.36	<0.001	0.314
Sex (females vs. males *)	0.573	0.449	0.00001
Interaction sex-country	15.00	<0.001	0.012
**Dependent variable: EHQ feelings score**			
Country (Poland vs. Lebanon *)	141.81	<0.001	0.107
Sex (females vs. males *)	0.032	0.858	0.00001
Interaction sex-country	14.68	<0.001	0.012

* Reference group. Numbers in bold indicate significant *p*-values.

**Table 7:** Relation between ON and sociodemographic variables and BMI ranges in Polish and Lebanese samples.

	DOS Sum Score	
	Polish Adults	Lebanese Adults
**Age**		
*r*, *p*	−0.030, 0.410	0.021, 0.638
*Z*, *p*	0.884, 0.188	
**Marital status**		
Single	17.68 ± 5.13	20.35 ± 7.42
Married	17.43 ± 4.89	20.10 ± 6.51
*p* marital status	0.402	
*p* country	**<0.001**	
*p* country * marital status	0.991	
**BMI categories**		
Underweight	16.33 ± 8.47	17.76 ± 8.43
Normal weight	17.56 ± 5.01	21.11 ± 7.35
Overweight	17.89 ± 5.54	19.74 ± 6.16
Obesity	18.36 ± 4.55	17.86 ± 6.11
*p* BMI category	0.112	
*p* country	**0.045**	
*p* country * BMI category	**0.008**	

* by (*p* country by marital status; *p* country by BMI category); *p*-values for statistically significant results are shown in bold.
